# Exosomal USP13 derived from microvascular endothelial cells regulates immune microenvironment and improves functional recovery after spinal cord injury by stabilizing IκBα

**DOI:** 10.1186/s13578-023-01011-9

**Published:** 2023-03-13

**Authors:** Xuhui Ge, Zheng Zhou, Siting Yang, Wu Ye, Zhuanghui Wang, Jiaxing Wang, Chenyu Xiao, Min Cui, Jiawen Zhou, Yufeng Zhu, Rixiao Wang, Yu Gao, Haofan Wang, Pengyu Tang, Xuhui Zhou, Ce Wang, Weihua Cai

**Affiliations:** 1 Department of Orthopedics, The First Affiliated Hospital of Nanjing Medical University, Nanjing, 210029 Jiangsu China; 2 Department of Emergency Medicine, The First Affiliated Hospital of Nanjing Medical University, Nanjing, 210029 Jiangsu China; 3 Department of Anesthesiology and Nursing, The First Affiliated Hospital of Nanjing Medical University, Nanjing, 210029 Jiangsu China; 4 Department of Human Anatomy, Nanjing Medical University, Nanjing, 211166 Jiangsu China; 5 Department of Pharmacology, China Pharmaceutical University, Nanjing, 211198 China; 6 Department of Orthopedics, Second Affiliated Hospital of Naval Medical University, Shanghai, 200003 China

**Keywords:** Exosomes, Microglia/macrophages polarization, Mitochondria, USP13/ IκBα, Spinal cord injury

## Abstract

Spinal cord injury (SCI) can result in irreversible sensory and motor disability with no effective treatment currently. After SCI, infiltrated macrophages accumulate in epicenter through destructed blood-spinal cord barrier (BSCB). Further, great majority of macrophages are preferentially polarized to M1 phenotype, with only a few transient M2 phenotype. The purpose of this study was to explore roles of vascular endothelial cells in microglia/macrophages polarization and the underlying mechanism. Lipopolysaccharide (LPS) was used to pretreat BV2 microglia and RAW264.7 macrophages followed by administration of conditioned medium from microvascular endothelial cell line bEnd.3 cells (ECM). Analyses were then performed to determine the effects of exosomes on microglia/macrophages polarization and mitochondrial function. The findings demonstrated that administration of ECM shifted microglia/macrophages towards M2 polarization, ameliorated mitochondrial impairment, and reduced reactive oxygen species (ROS) production in vitro. Notably, administration of GW4869, an exosomal secretion inhibitor, significantly reversed these observed benefits. Further results revealed that exosomes derived from bEnd.3 cells (Exos) promote motor rehabilitation and M2 polarization of microglia/macrophages in vivo. Ubiquitin-specific protease 13 (USP13) was shown to be significantly enriched in BV2 microglia treated with Exos. USP13 binds to, deubiquitinates and stabilizes the NF-κB inhibitor alpha (IκBα), thus regulating microglia/macrophages polarization. Administration of the selective IκBα inhibitor betulinic acid (BA) inhibited the beneficial effect of Exos in vivo. These findings uncovered the potential mechanism underlying the communications between vascular endothelial cells and microglia/macrophages after SCI. In addition, this study indicates exosomes might be a promising therapeutic strategy for SCI treatment.

**Supplementary Information:**

The online version contains supplementary material available at 10.1186/s13578-023-01011-9.

## Background

Spinal cord injury (SCI) is a central nervous system (CNS) disease characterized by long-term sensory and motor disorders. Due to high morbidity and mortality, SCI poses a major health burden worldwide [1, 2]. Primary injury of spinal cord is mainly related to injury of neurons and axons directly, whereas secondary injury is mainly caused by neuroinflammation that may result in cavitation and edema [3, 4]. The blood-spinal cord barrier (BSCB) or blood-brain barrier (BBB) is broken within 5 min after SCI, resulting in inflammatory response, excessive edema, progressive neuronal death, axonal dieback and glia activation. BSCB comprises continuous endothelial cells connected by molecular junctions physiologically, and functions as a barrier to suppress paracellular and transcellular transport. Notably, peripheral inflammatory cells, factors and other harmful cytokines infiltrate and accumulate in the lesion epicenter through the broken BSCB after SCI [5, 6]. In addition, activated resident microglia and infiltrated macrophages accumulate in the epicenter, resulting in a severer inflammatory response. Activated microglia and infiltrated macrophages can polarize to classical pro-inflammatory (M1) or alternative anti-inflammatory (M2) phenotype due to diverse signals in SCI microenvironment. Studies reported that M1 microglia/macrophages are harmful and M2 polarization promotes neurogenesis after SCI [7–9]. Therefore, studies should investigate the underlying mechanism regarding the cell crosstalk in the SCI microenvironment and shift the microglia/macrophages phenotype balance from M1 to M2 thus inhibiting detrimental neuroinflammation. We previously reported that a large number of microglia/macrophages surround the newborn blood vessels in the injured area after spinal cord injury, and microglia/macrophages play a crucial role in the tight junction formation of vascular endothelial cells through exosomal transfer of miR-155 [10]. However, whether vascular endothelial cells affect microglia/macrophages polarization and the role of its exosomes in microglia/macrophages is still unclear.

Exosomes are a kind of nanosized extracellular vesicles with a particle diameter of 50–150 nm. They are crucial elements of the paracrine secretions and implicated in mediation of the communications between different cells via transfer of genetic material messages including non-coding RNAs, mRNAs as well as proteins and preventing them from degrading [11, 12]. Several studies have explored the important role of exosomes in different biological process including regulation of intercellular signaling, angiogenesis, neurogenesis, inflammation and tumor progression [13–15]. However, only a few studies have explored interactions and communications between diverse cells in the microenvironment of SCI. Interactions and detailed mechanisms between the vascular endothelial cells and macrophages/microglia should be explored.

This current study demonstrates that exosomes derived from microvascular endothelial cells promote motor rehabilitation and M2 polarization of microglia/macrophages through transferring ubiquitin-specific protease 13 (USP13) post-injury. The delivered exosomal USP13 regulates microglia/macrophages polarization after SCI by stabilizing IκBα thus inhibiting NF-κB signaling pathway. Our work elucidates the potential mechanism between vascular endothelial cells and microglia/macrophages, which may contribute to a better understanding of the regulation in the microenvironment after SCI. Moreover, these findings support that exosomes derived from vascular endothelial cells are promising therapeutic agents for treatment of SCI.

## Materials and methods

### SCI model and treatment

All animals were raised and housed under the guidelines of the Animal Committee of the First Affiliated Hospital of Nanjing Medical University. SCI model was performed as previously described [16]. Isoflurane inhalation was used to anesthetize mice, and then laminectomy was carried out at T8 level to expose spinal cord. We established a mice SCI model via dropping a 5 g rod from a height of 6.5 cm to the spinal cord using spinal cord impactor (RWD). The muscles and skin were sutured immediately after injury. The mice bladders were evacuated 3 times a day until the bladder function was restored.

Mice subjected to SCI were randomly divided into several groups and administrated with bEnd.3 cells derived exosomes (Exos) or USP13-deleted exosomes (shUSP13-Exos) or corresponding control exosomes (shNC-Exos, 200 µg total protein of exosomes in 200 µL PBS), or an equal volume of PBS (200 µL) through the tail-vein injection post-injury as described previously [17, 18].

### Functional behavioral analysis

Mice were housed in a 12-h light-dark cycle and provided with food and water ad libitum. Prior to performing behavioral tests, all animals were acclimatized to the testing room or apparatus for 1 h.

The Basso Mouse Scale (BMS) score was evaluated by two investigators blinded to the groups at 1, 3, 7, 14, and 28 days post-injury according to the hindlimb locomotor function.

A rotarod test was carried out to evaluate balance and motor coordination after SCI. Mice were placed on an accelerating rotarod from 0 to 40 r.p.m. Each mouse was allowed to practice for one trial before the two test trials. The interval between each trial is 20 min. Time taken to fall was averaged from two individual test for a final score per mouse.

The (motor evoked potentials) MEPs were examined 28 days after SCI by electromyography test. The stimulation, recording, reference, and grounding electrode were placed at the rostral ends of spinal cord, flexor of biceps femoris, distal tendon of hindlimb muscle, and under skin, respectively. A single stimulation (0.5 mA, 0.5 ms, 1 Hz) was used to induce MEPs, then amplitude and latency were quantified to determine the hindlimb nerve conduction function.

### Immunofluorescence staining

The spinal cords of the injured areas were dissected and were then fixed in 4% paraformaldehyde overnight, followed by gradient dehydration in 15% and 30% sucrose solutions. Samples were then embedded in OCT compound and dissected into 14-µm thick sections. The frozen sections were washed with PBS and blocked with 5% BSA plus 0.3% Triton X-100 at room temperature for 1 h, and then incubated with primary antibodies at 4 ℃ overnight. Conjugated secondary antibodies were then used and DAPI was added. The images were photographed under a confocal microscope.

For cultured cell staining, the cells were fixed in 4% paraformaldehyde and permeabilized with 0.3% Triton X-100. After that, cells were blocked with 5% BSA for 1 h at room temperature and incubated with primary antibodies overnight at 4 ℃. Conjugated secondary antibodies were then used and DAPI was added. The images were photographed under a confocal microscope.

### Intralumbar delivery

Betulinic acid (BA) was administered through intralumbar injection as described previously [19, 20]. A 30-gauge beveled needle was connected to a 10 µL syringe and the needle was inserted between L4 and L5. 2 µg BA in 5 µL 0.1% DMSO (diluted in normal saline) was injected in the intralumbar using a syringe pump. Administration was carried out for more than 5 min to allow diffusion, and the cannula was left in place for 5 min. Administration of BA was performed twice a week and each mouse was given a total of 4 doses.

### Cell culture and transfection

bEnd.3 vascular endothelial cell line, BV2 microglia cell line, RAW264.7 macrophage cell line and HEK 293T cell line were purchased from Cell Bank of the Chinese Academy of Science. Lipopolysaccharide (LPS, 1 µg/ml) was added to stimulate BV2 microglia and RAW264.7 macrophages for 24 h followed by co-culturing with exosomes (200 µg/ml) of indicated groups. Cells were transfected using Lipofectamine 3000 reagents according to the manufacturer’s instructions.

Primary microglial cells were prepared as previously described [17]. Brain tissues of neonatal mouse were cut into 1 mm^3^ pieces and incubated with 0.125% trypsin (Gibco, USA) with gentle shaking at 37 ℃ for 10 min. The digested tissues were filtered with a 100-µm nylon mesh and cell suspension were then cultured in T75 flasks pre-coated with ploy-l-lysine (Beyotime, China) to obtain primary mixed glial cells. After 14 days of culture in vitro, the mature microglial cells were separated by shaking at 200 rpm for 2 h at room temperature for immunofluorescence staining.

### The shRNA and plasmid construction

The shRNA-control and shUSP13 were constructed by Genebay Biotech (Nanjing, China). The Flag-tagged USP13 (either WT or C345A mutant) expressing plasmid, Myc-tagged IκBα expression plasmid, and a series of HA-tagged Ubiquitin expression plasmids were generated by cloning their open reading frame with the N-terminal tag sequence into the vectors (Genebay Biotech, China). Scrambled lentiviral construct was used as a negative control.

### Exosome isolation and identification

Once bEnd.3 cells achieving 80% confluency, the culture medium was replaced with exosome-free fetal bovine serum for 48 h. The medium was then acquired and centrifuged at 300×*g* for 10 min and 2000×*g* for 20 min at 4 ℃. Supernatant was collected and centrifuged at 10,000×*g* for 30 min followed by filtered using a 0.22-µm sterile filter (Steritop™ Millipore). For exosomes collection and purification, filtered medium was subsequently ultra-centrifugated at 100,000×*g* for 60 min at 4 ℃. Then the supernatant was discarded and sterile PBS was used to wash the pellet of exosomes, and another ultracentrifugation procedure (100,000×*g*, 60 min) was continued. Exosomes were then resuspended, aliquoted and stored under − 80 °C or used immediately for downstream experiments.

Size distribution of exosomes was explored using Nanoparticle Tracking Analysis (NTA). Morphological analysis of exosomes was performed using a transmission electron microscope (TEM).

The detection of exosomes uptake in BV2 microglia was performed using exosomes-containing PBS medium which was incubated with Dil solution. The images were photographed under a confocal microscope.

### In vitro detection of USP13 transfer

To detect the direct transfer of exosomal USP13, bEnd.3 cells were transfected with a GFP-USP13 fusion mRNA construct (Genebay biotech, China), after which exosomes were purified and applied to BV2 microglia as previously reported [21]. Following incubation, BV2 cells were fixed with 4% PFA and permeabilized with 0.3% Triton X-100, and stained with DAPI. The GFP fluorescence in the target BV2 cells were observed under a confocal microscope.

### Quantitative real-time PCR (qRT-PCR)

Total RNA was collected using TRIzol reagent (Invitrogen, USA). The RNA was then reverse transcribed into cDNA using a PrimeScript RT Reagent Kit (Takara, Japan). qRT-PCR was performed using SYBR Green PCR master mix. Relative expression levels of target genes were normalized to GAPDH and quantified using the 2^−ΔΔCT^ method.

### RNA sequence (RNA-seq)

RNA-seq analysis was performed by Genminix Information Co., Ltd., (Shanghai, China). Total RNAs from BV2 microglia treated with Exos and PBS (n = 3/group) were extracted. Quality RNA samples were converted into cDNA libraries following previously described methods [22]. Following fragments purification, the purified products were amplified with 12–15 cycles of PCR to create the final cDNA library. Finally, Libraries were sequenced on the Illumina Hiseq X Ten following the manufacturer’s protocols. Data analysis was performed in R studio software after normalization, log_2_ transformation and probe annotation. Fold changes > 1.5 and P < 0.05 represented differentially expressed genes (DEGs).

### Flow cytometry

Flow cytometry was used to evaluate the polarization of BV2 microglia and RAW264.7 macrophages. Cells in indicated groups were collected and incubated with specific antibodies (F4/80, iNOS and CD206) according to the manufacturer’s instructions.

### Oxygen consumption rate (OCR) measurement

Measurement of OCR was determined using a Seahorse XF96 Metabolic Flux Analyzer as described previously [10]. OCR was determined by addition of 2 µM oligomycin, 1 µM carbonyl cyanide 4-(trifluoromethoxy) phenylhydrazone (FCCP), 1 µM antimycin A as well as 1 µM rotenone (A&R) sequentially. XFe Data was determined using wave software (Seahorse Biosciences, USA). The basal respiration, ATP production, respiratory capacity and respiratory reserve were determined following the manufacturer’s protocol.

### ROS and mitochondrial membrane potential evaluation

ROS detection Kit (Beyotime, China) was used to determine ROS levels using 2′,7′-dichlorofluorsecein-diacetate (DCFH-DA) by flow cytometry analysis. Mitochondrial membrane potential was examined by JC-1 evaluation Kit (Beyotime, China) and quantified by aggregate-to-monomer fluorescence intensity ratio.

### Western blot

Cells and spinal cord samples were lysed using RIPA lysis buffer. Equal amounts of protein were separated by SDS-PAGE, transferred to PVDF membranes, blocked with 5% BSA and incubated with specific primary antibodies at 4 ℃ overnight. Corresponding secondary antibodies were then incubated with membranes for 2 h followed by enhanced chemiluminescent reagent treatment. The expression of protein was evaluated using Image J software (National Institutes of Health, USA).

### Immunoprecipitation (IP)

RIPA lysis buffer containing protease inhibitors (Beyotime, China) were used to collect and lyse cells. The protein concentration was determined using bicinchoninic acid (BCA) assay (Thermo Fisher, USA). Control IgG together with 20 µL protein A/G plus-agarose (Santa Cruz Biotechnology, USA) were used to preclear cell lysates for 1 h followed by immunoprecipitated with specific antibodies and protein A/G plus-agarose at 4 ℃ overnight. After that, immunocomplexes were collected and washed five times with lysis buffer and boiled. At last, the bound protein was separated by SDS-PAGE for immunoblotting.

### In vivo ubiquitylation assays

Endogenous IκBα ubiquitination was determined by immunoprecipitation with anti-IκBα antibody firstly followed by immunoblotted with anti-ubiquitin antibody. Ubiquitylation of exogenous IκBα was evaluated after transfection of Flag-tagged USP13 (WT or C345A mutant), Myc-tagged IκBα and HA-tagged ubiquitin in HEK 293T cells. Lysate proteins were precipitated and evaluated by immunoblotted with specific antibodies.

### Statistical analysis

All data are shown as mean ± standard deviation, and statistical analysis was performed in GraphPad Prism (version 8.0, GraphPad Software Inc., USA). Student’s t-test was used for comparisons between two groups, and one-way or two-way ANOVA followed by the post-hoc Tukey correction was used for multivariate analysis. P value < 0.05 was considered to be significant.

## Results

### Identification and characterization of exosomes derived from vascular endothelial cells

To elucidate the potential role of exosomes in the crosstalk between vascular endothelial cells and microglia/macrophages in SCI microenvironment, exosomes of microvascular endothelial cell line bEnd.3 cells were extracted, characterized and named Exos. TEM images showed that exosomes had cup-shaped or spherical morphology approximately 100 nm in size (Additional file 1: Fig. S1a). NTA results were used to generate the distribution curve of the nanoparticle size of exosomes (Additional file 1: Fig. S1b). Western blot assay was performed to confirm the exosomal surface markers (Additional file 1: Fig. S1c). Moreover, a Dil dye-labelled exosomes was applied to co-culture with target BV2 microglia for 24 h. Images showed that exosomes were present in cytoplasm, indicating successful uptake of exosomes by target cells (Additional file 1: Fig. S1d).

### Exos treatment promotes M2 polarization and ameliorates mitochondrial impairment of LPS-pretreated microglia/macrophages in vitro

**Fig. 1:**
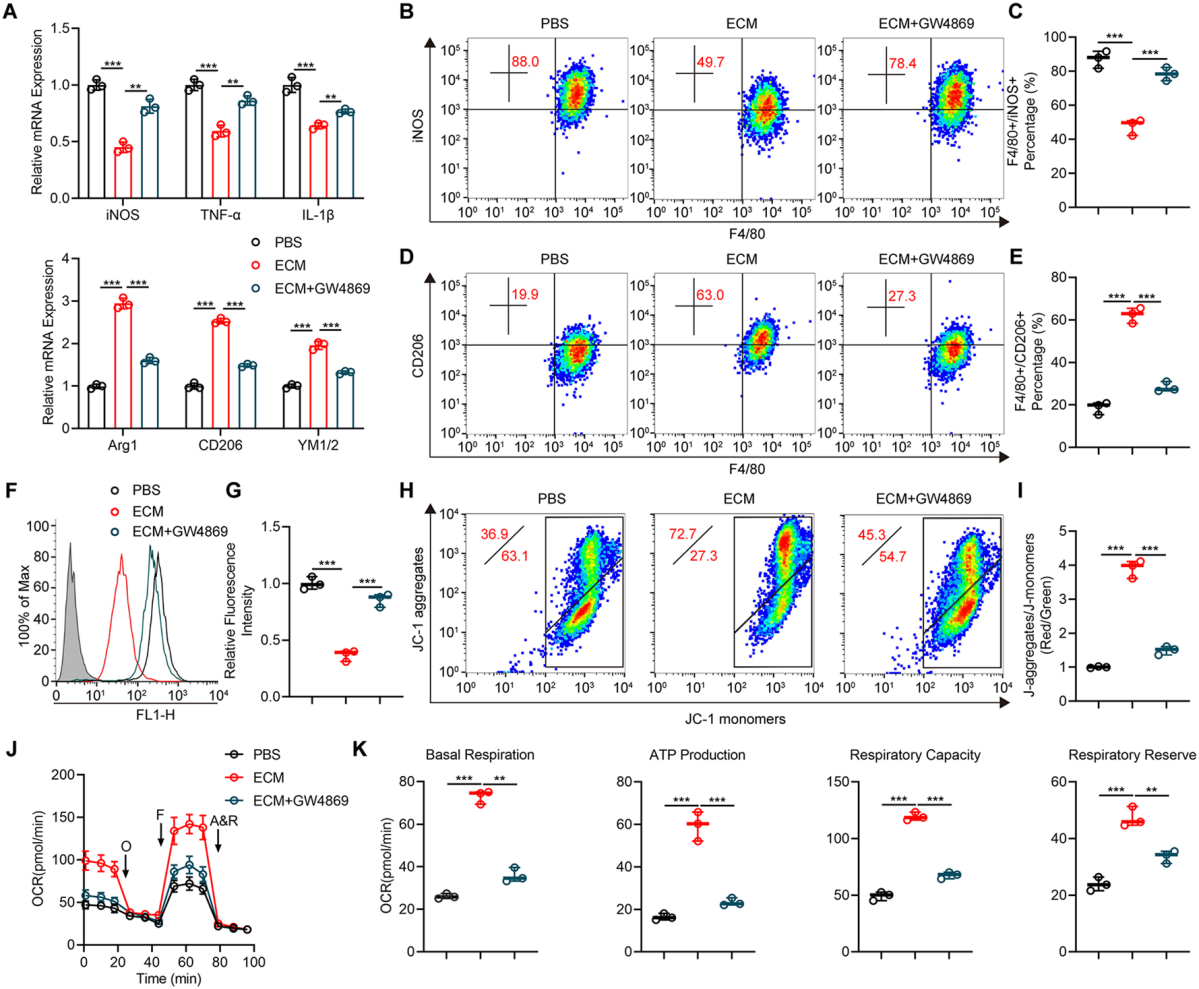
Exos treatment promotes M2 polarization and ameliorates mitochondrial impairment of LPS-pretreated microglia/macrophages in vitro. **a** Detection of mRNA expression of M1 and M2 markers in indicated groups. **P < 0.01, ***P < 0.001. **b**, **c** Detection and quantification of microglia M1 polarization by flow cytometry. ***P < 0.001. **d**, **e** Measurement and quantification of microglia M2 polarization by flow cytometry. ***P < 0.001. **f**, **g** Flow cytometry detection and quantification of ROS level. ***P < 0.001. **h**, **i** Detection and quantification of mitochondrial potential by JC-1 staining. ***P < 0.001. **j**, **k** Measurement of OCR and quantification of basal respiration, ATP production, respiratory capacity and respiratory reserve. **P < 0.01, ***P < 0.001

Microglia and macrophages are markers of SCI pathology and exert corresponding functions in response to neuroinflammation that subsequently contribute to secondary injury. Therefore, the role of exosomes in altering the polarization of LPS-pretreated microglia/macrophages was explored. bEnd.3 cells were cultured and endothelial cells conditional medium (ECM) was obtained. To further explore the underlying effects of bEnd.3 cells on microglia, BV2 microglia pretreated with LPS for 24 h was cultured with ECM. The findings showed that administration of ECM significantly decreased expression levels of M1 markers (iNOS, TNF-α, IL-1β) whereas increased expression levels of M2 markers (Arg1, CD206, YM1/2) (Fig. 1a). Moreover, flow cytometry analysis demonstrated a decrease of F4/80^+^ iNOS^+^ cells as well as an increase of F4/80^+^ CD206^+^ cells in ECM group compared to corresponding control (Fig. 1b–e). Given that exosomes are implicated in paracrine effects between cell crosstalk, and to further explore if bEnd.3 cells regulated microglia M2 polarization through exosomes, bEnd.3 cells were pretreated with GW4869, which is an exosomal secretion inhibitor. We further confirmed that inhibition of exosomal secretion obviously reversed ECM-mediated microglia M2 polarization (Fig. 1a–e). We also performed above related experiments in RAW264.7 macrophages and primary microglial cells and found similar results (Additional file 1: Fig. S2a–f). These findings show that exosomes might be involved in cell-to-cell communications between vascular endothelial cells and microglia/macrophages and are implicated in vascular endothelial cells-induced M2 polarization in microglia/macrophages in vitro.

Several studies have investigated the link between microglia/macrophages polarization and mitochondrial functions [23, 24]. In the present study, we further investigated ROS levels which is also another M1 microglia/macrophages feature. The findings showed that ECM administration significantly reduced ROS level in BV2 microglia and this anti-ROS effect was abolished by inhibition of exosomal secretion (Fig. 1f, g). JC-1 staining results showed that mitochondrial potential was significantly restored after treating cells with ECM, and this beneficial effect was partly inhibited by administration of GW4869 (Fig. 1h, i). Moreover, ECM administration significantly increased OCR which is an oxidative phosphorylation biomarker, however, GW4869 administration downregulated OCR expression (Fig. 1j). In addition, mitochondrial activities including basal respiration, ATP production, respiration capacity, and respiration reverse in BV2 microglia were markedly upregulated by ECM administration but were reversed by addition of GW4869 (Fig. 1k).

### Exos treatment promotes better functional recovery post-injury in vivo

**Fig. 2:**
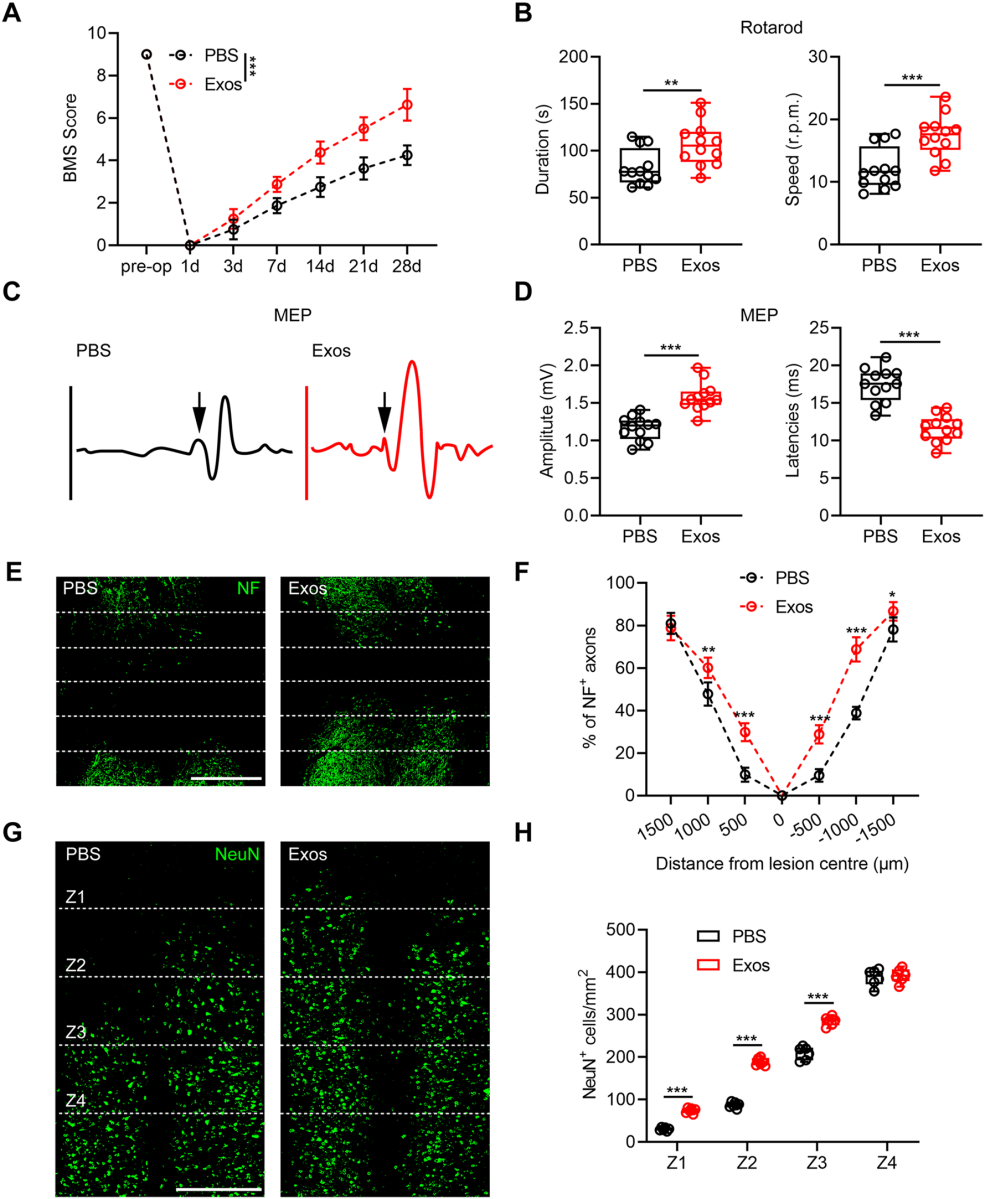
Exos treatment promotes better functional recovery post-injury in vivo. **a** BMS scores in the course of the 28-day recovery period in indicated groups. ***P < 0.001. **b** Evaluation of motor recovery by rotarod tests at day 28 after SCI. **P = 0.0086, ***P = 0.0008. **c**, **d** Representative images and quantification of MEP amplitude and latencies in indicated groups at day 28 post-injury. ***P < 0.001. **e**, **f** Representative immunofluorescence staining of NF in spinal cords at day 28 post-injury and quantification of NF^+^ area to the total area of uninjured axons at indicated distances from the SCI lesion core (Scale bar = 1000 μm). *P = 0.036, **P = 0.0069, ***P < 0.001. **g**, **h** Immunostaining of NeuN in spinal cords at day 28 post-injury and quantification of viable neurons in Z1–Z4 zones adjacent to lesion core (Scale bar = 1000 μm). ***P < 0.001

To further explore the functions of vascular endothelial cells in SCI microenvironment and their potential work on microglia/macrophages, Exos were extracted and injected immediately post-injury. Functional motor analyses were performed at indicated timepoint. BMS score after SCI indicated that mice administered with Exos had markedly greater motor recovery in the course of the 28-day recovery period in contrast to those administered with PBS (Fig. 2a). Moreover, results from rotarod testing showed better functional motor rehabilitation in Exos group in comparison with the control group (Fig. 2b). Furthermore, electrophysiological analyses showed that MEPs in mice administered with Exos exhibited higher amplitude as well as shorter latency (Fig. 2c, d). These findings on behavioral tests indicate that administration of Exos promotes functional motor rehabilitation in mice post-injury.

Also, axons adjacent the injured area was further investigated. Neurofilament positive (NF^+^) axons were assessed for evaluation of axonal regeneration. A considerably greater quantity of NF^+^ axons was found in Exos group at day 28 post injury compared to PBS group (Fig. 2e, f). NeuN, which is the neuronal marker, was applied to represent viable neurons in particular areas (Z1–Z4) located at different distances as previous described [16, 25]. Administration of Exos caused an obvious increase in number of NeuN^+^ neurons in the Z1–Z3 zones compared to the PBS group (Fig. 2g, h). These findings demonstrate that Exos administration promotes functional recovery and axonal regeneration in mice after SCI.

### Exos treatment promotes M2 polarization of microglia/macrophages in vivo

**Fig. 3:**
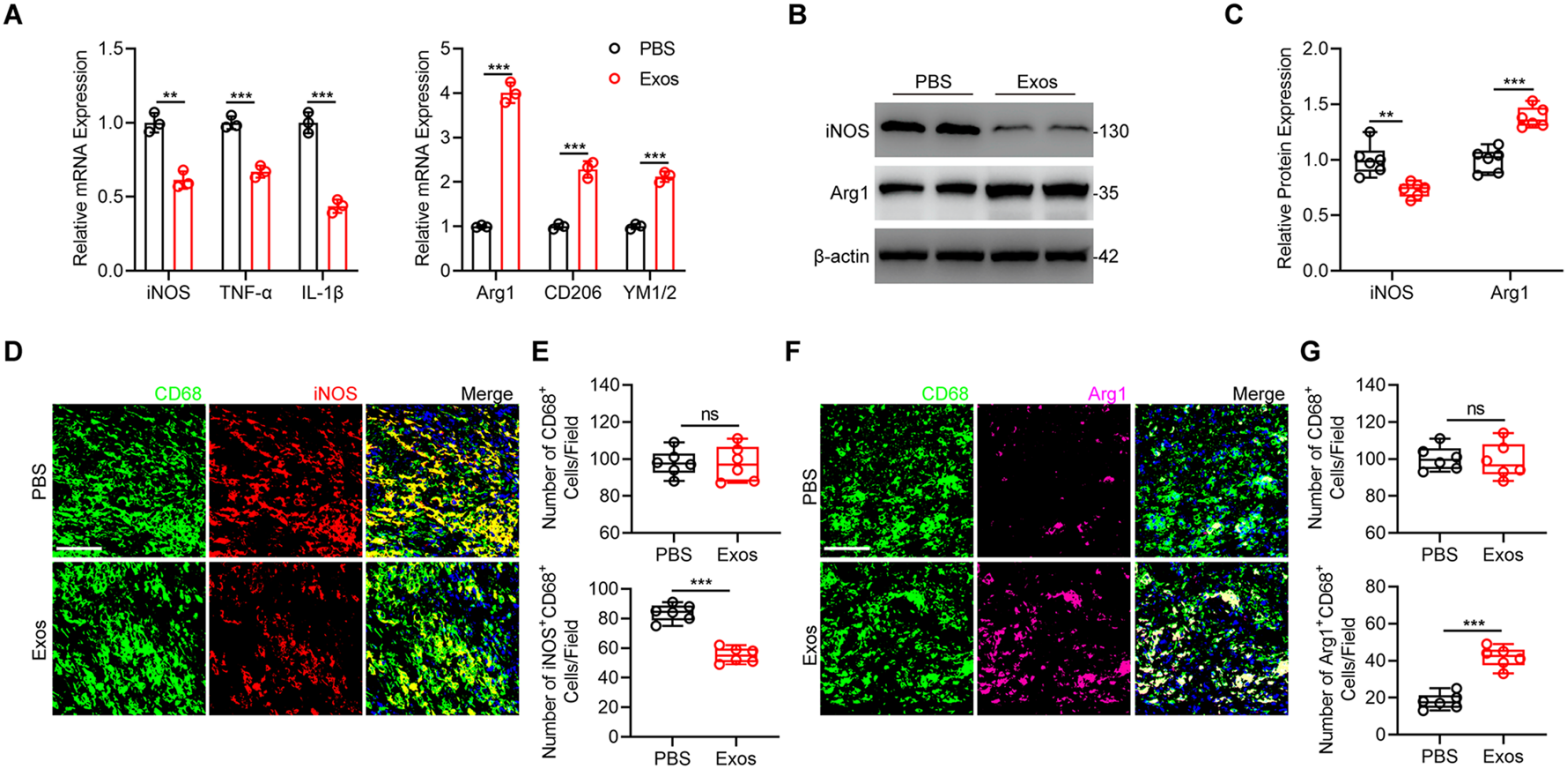
Exos treatment promotes M2 polarization of microglia/macrophages in vivo. **a** Detection of mRNA levels of M1 and M2 markers in injured spinal cords at day 7 post-injury.**P = 0.0016, ***P < 0.001. **b**, **c** Detection and quantification of iNOS and Arg1 protein expression level at day 7 post-injury. **P = 0.0058, ***P = 0.0005. **d**, **e** Double-staining of CD68 and iNOS at day 7 after SCI and quantification of CD68^+^ cells and M1 microglia/macrophages (Scale bar = 100 μm). ***P = 0.0003. **f**, **g** Double-staining of CD68 and Arg1 at day 7 after SCI and quantification of CD68^+^ cells and M2 microglia/macrophages (Scale bar = 100 μm). ***P = 0.0007

Expression level of M1 markers in Exos group was significantly decreased whereas M2 markers increased compared with PBS group (Fig. 3a). In addition, western blot analysis showed that lower protein levels of M1 markers and higher protein levels of M2 markers in the Exos group (Fig. 3b, c). Moreover, we used CD68, which represents activated microglia/macrophages, together with iNOS or Arg1 respectively to explore the M1/M2 microglia/macrophages polarization via double immunofluorescence staining in indicated groups post-injury. No significant difference was found in quantification of CD68^+^ cells (Fig. 3d–g). However, an obvious reduction in M1 microglia/macrophages and an obvious increase in M2 microglia/macrophages were observed after administration of Exos compared with the PBS group at day 7 post-injury in vivo (Fig. 3d–g). These findings indicate that Exos play key role in regulating microglia/macrophages M1/M2 polarization in vivo.

### Exos treatment promotes functional rehabilitation and M2 polarization of microglia/macrophages via delivering USP13 post-injury

**Fig. 4:**
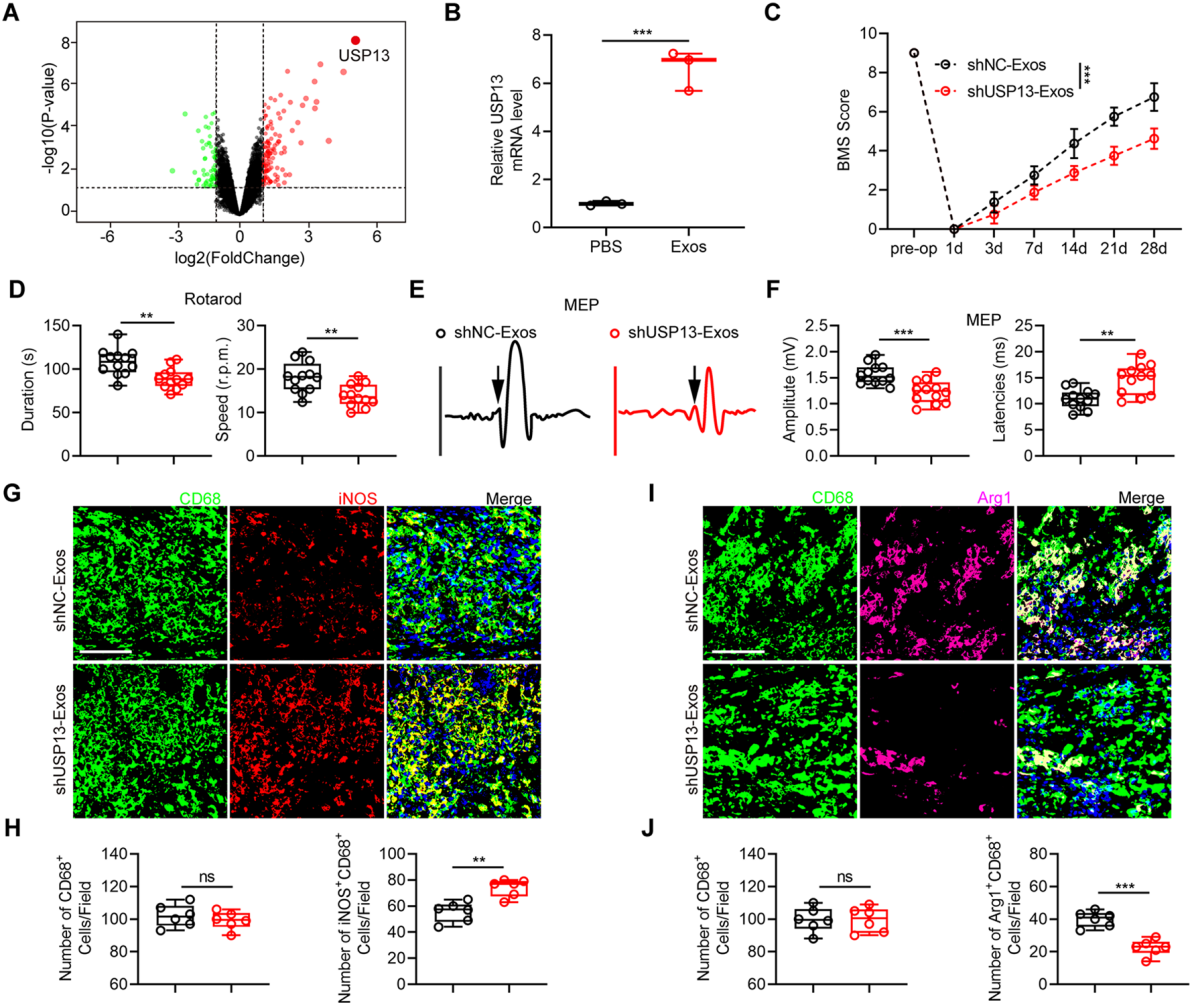
Exos treatment promote functional rehabilitation and M2 polarization of microglia/macrophages post-injury by delivering USP13. **a** Volcano plot of genes of BV2 microglia treated with PBS and Exos. Red and green dots represent up- and down-regulated DEGs, respectively. **b** mRNA expression level of USP13 in BV2 microglia treated with PBS and Exos. ***P = 0.0003. **c** BMS scores in the course of the 28-day recovery period in indicated groups. ***P < 0.001. **d** Evaluation of motor recovery by rotarod tests at day 28 after SCI. **P < 0.01. **e** Assessment of electromyography using MEP at day 28 after SCI. **f** Quantification of MEP amplitudes and latencies in indicated groups. **P = 0.0010, ***P = 0.0005. **g**, **h** Double-staining of CD68 and iNOS at day 7 after SCI and quantification of CD68^+^ cells and M1 microglia/macrophages (Scale bar = 100 μm). **P = 0.0012. **i**, **j** Double-staining of CD68 and Arg1 at day 7 after SCI and quantification of CD68^+^ cells and M2 microglia/macrophages (Scale bar = 100 μm). ***P < 0.001

Exosomes contain mRNAs, therefore, exosomal mRNAs may exert biological beneficial functions in vitro and in vivo as observed above in this study. To explore the mechanism underlying Exos functions, RNAs were extracted from BV2 microglia treated with Exos or PBS, and RNA-seq was performed. Analysis showed that the deubiquitinase USP13 was the most significantly upregulated mRNA in BV2 microglia treated with Exos (Fig. 4a). To verify the USP13 mRNA profile data, expression level of USP13 was analyzed and confirmed by qRT-PCR in vitro (Fig. 4b).

To further explore the role of exosomal USP13, lentiviral-based methods were used to knockdown USP13 and the corresponding negative control in bEnd.3 cells (Additional file 1: Fig. S3a). Exosomes were then isolated followed by co-cultured with target BV2 microglia. USP13 was significantly silenced in shUSP13-Exos in comparison with shNC-Exos (Additional file 1: Fig. S3b). Moreover, USP13 was found to be obviously decreased in BV2 microglia treated with shUSP13-Exos (Additional file 1: Fig. S3c). Additionally, robust GFP fluorescence was observed in BV2 microglia incubated with exosomes from plasmid-loaded bEnd.3 cells, whereas no detectable fluorescence was found in microglia treated with exosomes from empty-vector bEnd.3 cells (Additional file 1: Fig. S3d). These results showed that exosomal USP13 was delivered to target BV2 microglia and could be associated with the beneficial functions of Exos post-injury.

Furthermore, shNC-Exos and shUSP13-Exos were administrated in vivo to explore the functional role of exosomal USP13 in Exos-regulated benefits after SCI. Analysis based on functional experiments as described above showed that mice administrated with shUSP13-Exos exhibited worse functional recovery compared with shNC-Exos (Fig. 4c–f). Moreover, administration of shUSP13-Exos markedly suppressed M2 microglia/macrophages polarization post-injury through qRT-PCR and western blot analysis (Additional file 1: Fig. S4a–c). The double-immunofluorescence experiments demonstrated consistent results (Fig. 4g–j). These findings indicate that Exos promote functional recovery and M2 polarization of microglia/macrophages via delivering USP13.

### Exos treatment regulates microglia M2 polarization and modulates mitochondrial function through transferring USP13 in vitro

shNC-Exos and shUSP13-Exos were administered to BV2 microglia to confirm the function of USP13. Silencing of USP13 in Exos evidently suppressed the M2 shift and upregulated ROS production in microglia in comparison with the control by qRT-PCR and flow cytometry analysis (Additional file 1: Fig. S5a–g). Notably, mitochondrial potential, OCR, and mitochondrial activities were decreased after administration of shUSP13-Exos (Additional file 1: Fig. S5h–k). These in vitro results demonstrate that exosomal USP13 could regulate microglia polarization and mitochondrial function.

### USP13 binds to and stabilizes IκBα

**Fig. 5:**
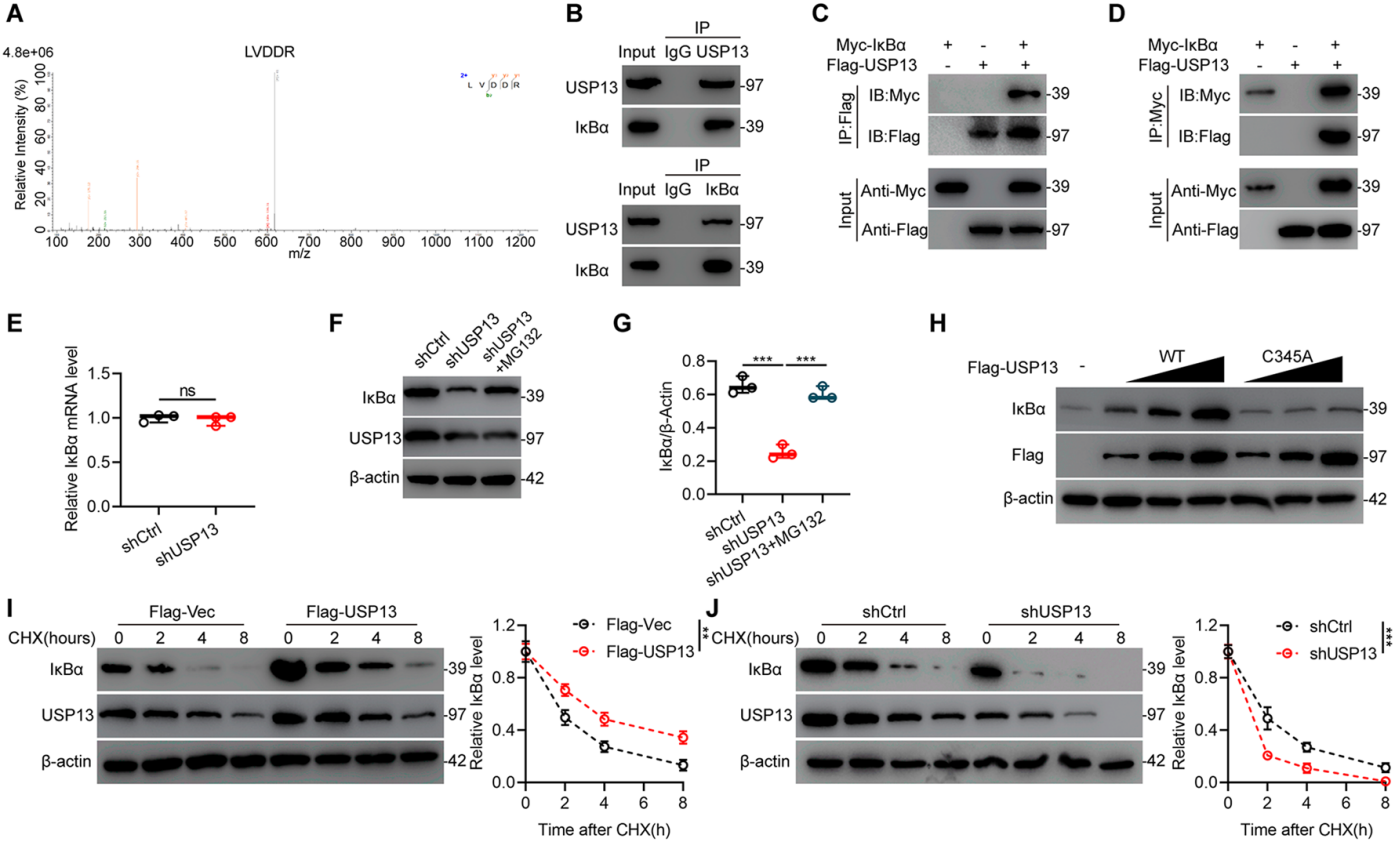
USP13 binds to and stabilizes IκBα. **a** Analysis by IP/MS indicated IκBα interacts with USP13. **b** Detection of endogenous protein interactions between USP13 and IκBα in BV2 microglia by Co-IP. **c**, **d** Detection of exogenous protein interactions between USP13 and IκBα in HEK 293T cells by Co-IP. Flag-tagged USP13 and Myc-tagged IκBα plasmids were transfected in HEK 239T cells. **e** IκBα mRNA level in indicated groups. **f**, **g** Detection and quantification of IκBα and USP13 protein level in indicated groups with or without proteasome inhibitor MG132 treatment by western blot analysis. ***P < 0.001. **h** Increasing amounts of Flag-tagged USP13 (WT or C345A mutant) were transfected and the expression levels of IκBα and Flag-tagged USP13 were detected by western blot analysis. **i**, **j** Measurement and quantification of IκBα protein expression in indicated groups with cycloheximide (CHX, 10 µg/ml) treatment by western blot analysis. **P = 0.0021, ***P < 0.001

To further explore the underlying mechanism in USP13-regulated microglia/macrophages polarization, IP coupled with mass spectrum (IP/MS) was performed to determine which protein interacts with USP13. IκBα, which is a key player in inhibiting NF-κB signaling pathway activation, was identified as a protein which interacts with USP13 (Fig. 5a). Co-immunoprecipitation (Co-IP) analysis showed that endogenous IκBα could be precipitated by USP13 antibody and endogenous USP13 was precipitated by IκBα antibody as well in BV2 microglia (Fig. 5b). Furthermore, the interaction between exogenous USP13 and IκBα was investigated and confirmed in HEK 293T cells (Fig. 5c, d). In all, above findings show that USP13 binds to IκBα.

USP13 binds to IκBα, therefore, the effect of downregulating USP13 in BV2 microglia on IκBα expression level was further investigated. As shown in Fig. 5e and Additional file 1: Fig. S6a, downregulation of USP13 significantly decreased IκBα protein level while had no significant effect on IκBα mRNA level. This result indicates that USP13 regulates the protein level instead of mRNA level of IκBα. Interestingly, addition of MG132 which is the proteasome inhibitor significantly reverse the decreased IκBα protein level when silencing of USP13 (Fig. 5f, g). Furthermore, knockdown of USP13 upregulated expression level of nuclear accumulation of p65 in BV2 microglia, implying that it activated the NF-κB signaling pathway (Additional file 1: Fig. S6a, b). Further analysis was performed to explore if IκBα can be stabilized by USP13. IκBα protein level was increased after overexpression of USP13, whereas the level was not influenced by overexpression of catalytically inactive C345A mutant USP13 (Fig. 5h). Next, we investigated the potential role of USP13 on the endogenous IκBα protein stability in the presence of protein synthesis inhibitor cycloheximide (CHX). As shown in Fig. 5i, j, USP13 overexpression significantly suppressed IκBα degradation while USP13 downregulation accelerated IκBα degradation.

**Fig. 6:**
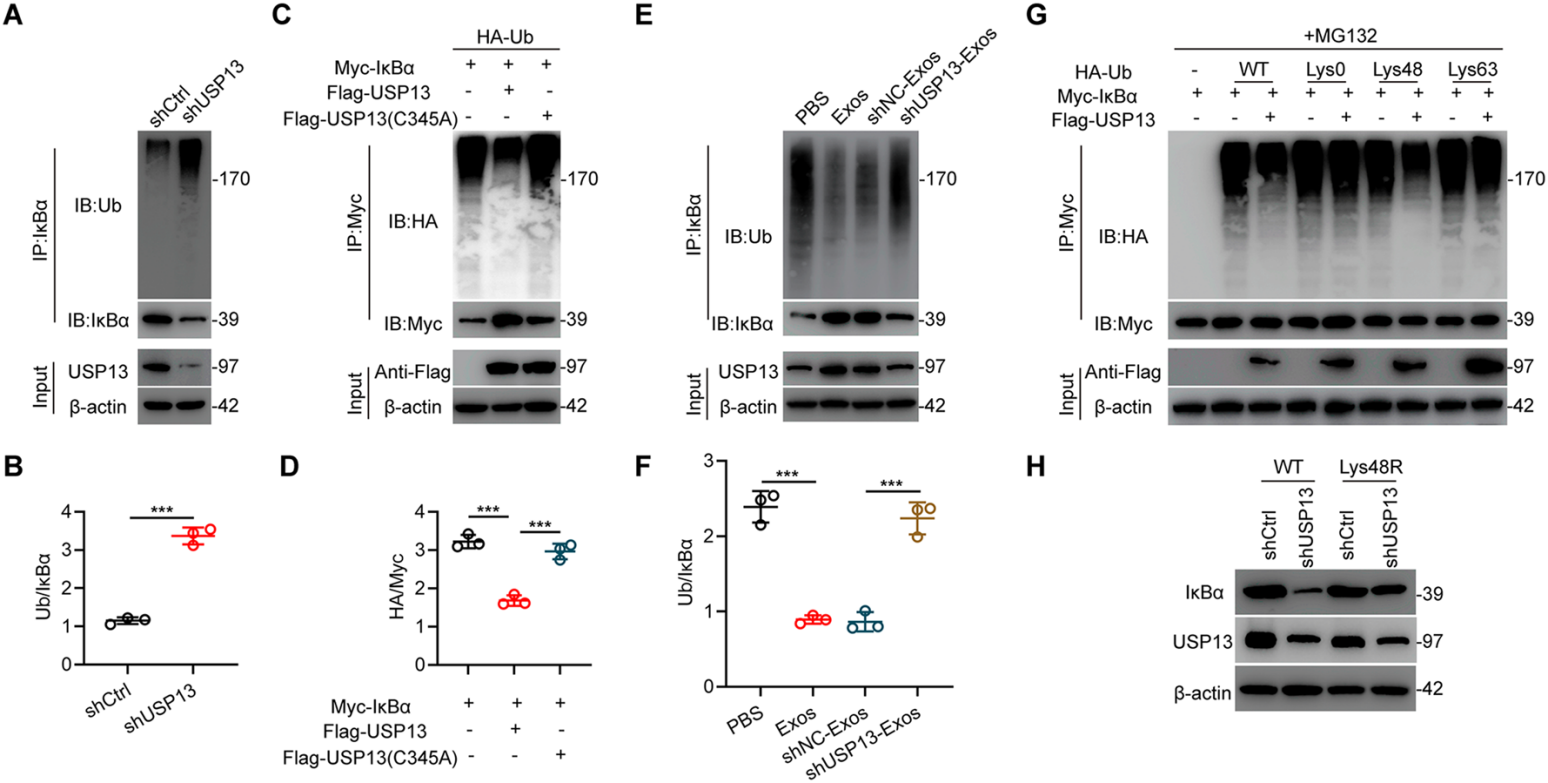
USP13 suppresses IκBα ubiquitination. **a**, **b** Evaluation and quantification of endogenous IκBα ubiquitination in indicated groups in BV2 microglia. ***P < 0.001. **c**, **d** Evaluation and quantification of exogenous IκBα ubiquitination in HEK 293T cells co-transfected with Flag-tagged USP13 (WT or C345A), HA-tagged Ub and Myc-tagged IκBα. ***P < 0.001. **e**, **f** Evaluation and quantification of IκBα ubiquitination in spinal cords of indicated groups. ***P < 0.001. **g** Evaluation of the IκBα ubiquitylation linkage in HEK 293T cells co-transfected with Flag-USP13, Myc-IκBα and the specific HA-Ub, Lys0, Lys48, or Lys63 plasmids. **h** Evaluation of IκBα and USP13 protein expression levels in HEK 293T cells transfected with Ub WT or Ub Lys48R in indicated groups

USP13 is a deubiquitinase (DUB) and regulates IκBα stability, therefore, analyses were performed to explore if USP13 regulates IκBα ubiquitination. Silencing of USP13 significantly enhanced the ubiquitination level of IκBα but decreased the protein level compared with shCtrl (Fig. 6a, b). To further confirm the role of USP13 in the regulation on IκBα ubiquitination, Flag-USP13 (either WT or C345A mutant), Myc-IκBα as well as HA-Ub were co-transfected in HEK 293T cells. Analysis showed that overexpression of WT USP13 decreased IκBα ubiquitination while transfection of C345A mutant USP13 showed no significant influence (Fig. 6c, d). Effect of USP13 on IκBα ubiquitination was further investigated in vivo. Notably, ubiquitination level of spinal cords IκBα was remarkably inhibited in Exos group compared with PBS group, whereas it was promoted in shUSP13-Exos group compared with corresponding control after SCI (Fig. 6e, f). It’s recognized that Lys48- and Lys63-linked chains are two main types of polyubiquitin chains. Therefore, the form of polyubiquitin modification of IκBα protein regulated by USP13 was explored. As shown in Fig. 6g, USP13 cleaved Lys48-linked polyubiquitin chains instead of Lys63-linked on IκBα protein. To further verify that Lys48-linked polyubiquitination is essential for USP13-mediated IκBα protein stability, we transfected a Lys48 mutant (Lys48R) type of ubiquitin in USP13-silenced HEK 293T cells. It was shown that transfection of Lys48R ubiquitin reversed the influence of silencing of USP13 in decreasing IκBα protein level (Fig. 6h). These findings indicate that USP13 regulates IκBα stability in microglia as well as injured spinal cord.

### Exosomal USP13 regulates microglia polarization and mitochondrial function through stabilizing IκBα

**Fig. 7:**
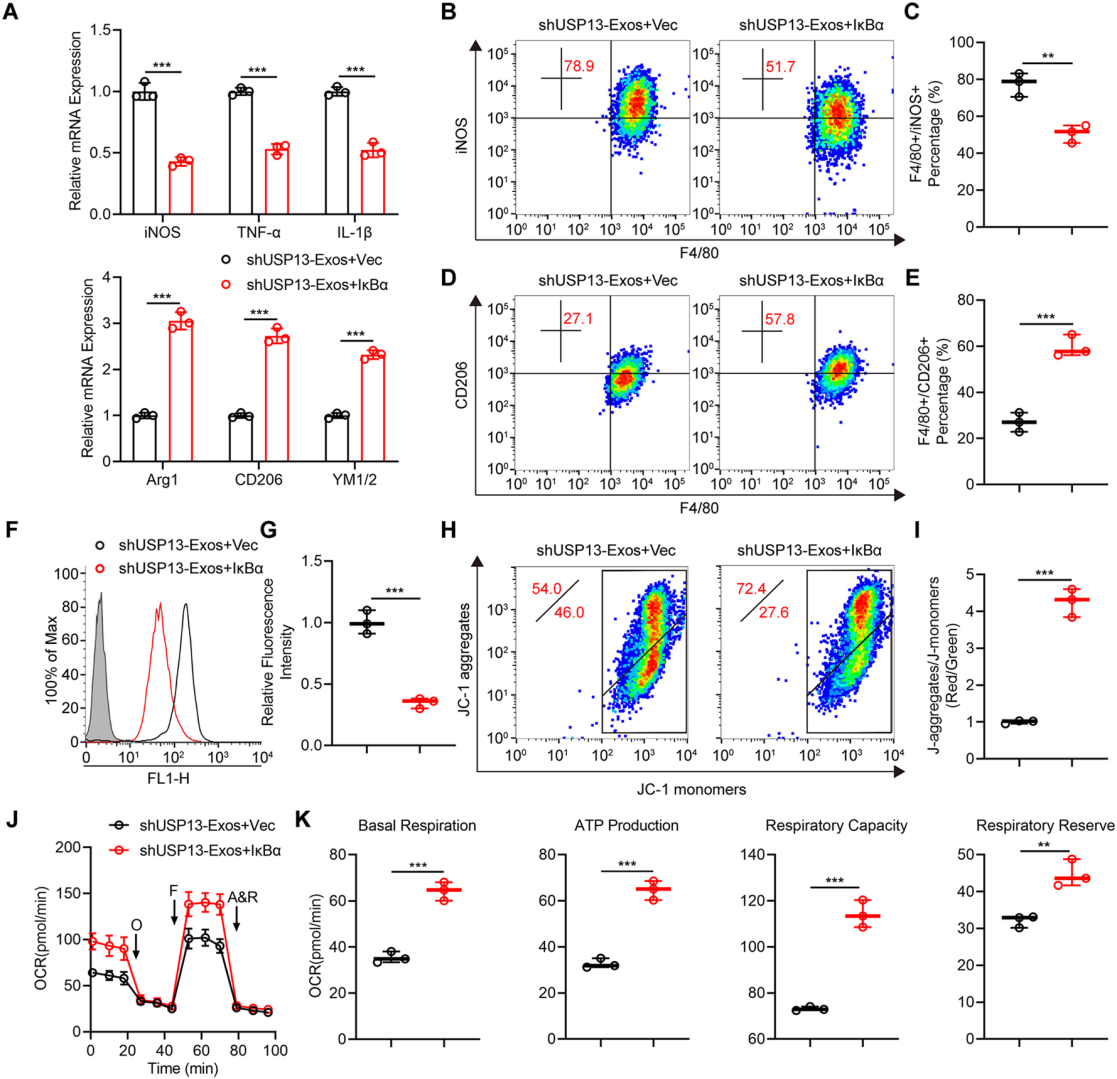
Exosomal USP13 regulates microglia polarization and mitochondrial function through stabilizing IκBα. **a–k** Rescue experiments were performed to explore the functions of exosomal USP13/IκBα in microglia. Rescue experiments for exosomal USP13 deletion were conducted by ectopic overexpressing IκBα. **a** Detection of mRNA expression of M1 and M2 markers in indicated groups. ***P < 0.001. **b–e** Examination and quantification of M1 and M2 microglia by flow cytometry in indicated groups. **P = 0.0043, ***P = 0.0009. (f, g) Flow cytometry measurement and quantification of ROS level in indicated groups. ***P = 0.0004. **h**, **i** Detection and quantification of mitochondrial potential by JC-1 staining. ***P = 0.0001. **j** Measurement of OCR. **k** Quantification of mitochondrial activities. **P = 0.0055, ***P < 0.001

Next, IκBα was overexpressed in BV2 microglia and in vitro analyses were performed to further investigate the potential relationship between exosomal USP13 and IκBα in regulating microglia polarization. As shown in Fig. 7a–e, results showed that ectopic IκBα overexpression significantly reversed microglia polarization caused by administration of shUSP13-Exos and promoted M2 polarization in BV2 microglia. Furthermore, overexpression IκBα decreased ROS production induced by administration of shUSP13-Exos (Fig. 7f, g). Also, downregulation in mitochondrial potential, OCR and mitochondrial activities after administration of shUSP13-Exos were reversed when overexpression of IκBα (Fig. 7h–k). These results show that exosomal USP13 stabilizes IκBα and thus regulates microglia polarization as well as mitochondrial function in vitro.

### Exos treatment promotes functional rehabilitation and M2 polarization of microglia/macrophages through stabilizing IκBα thus inhibiting NF-κB signaling activation

**Fig. 8:**
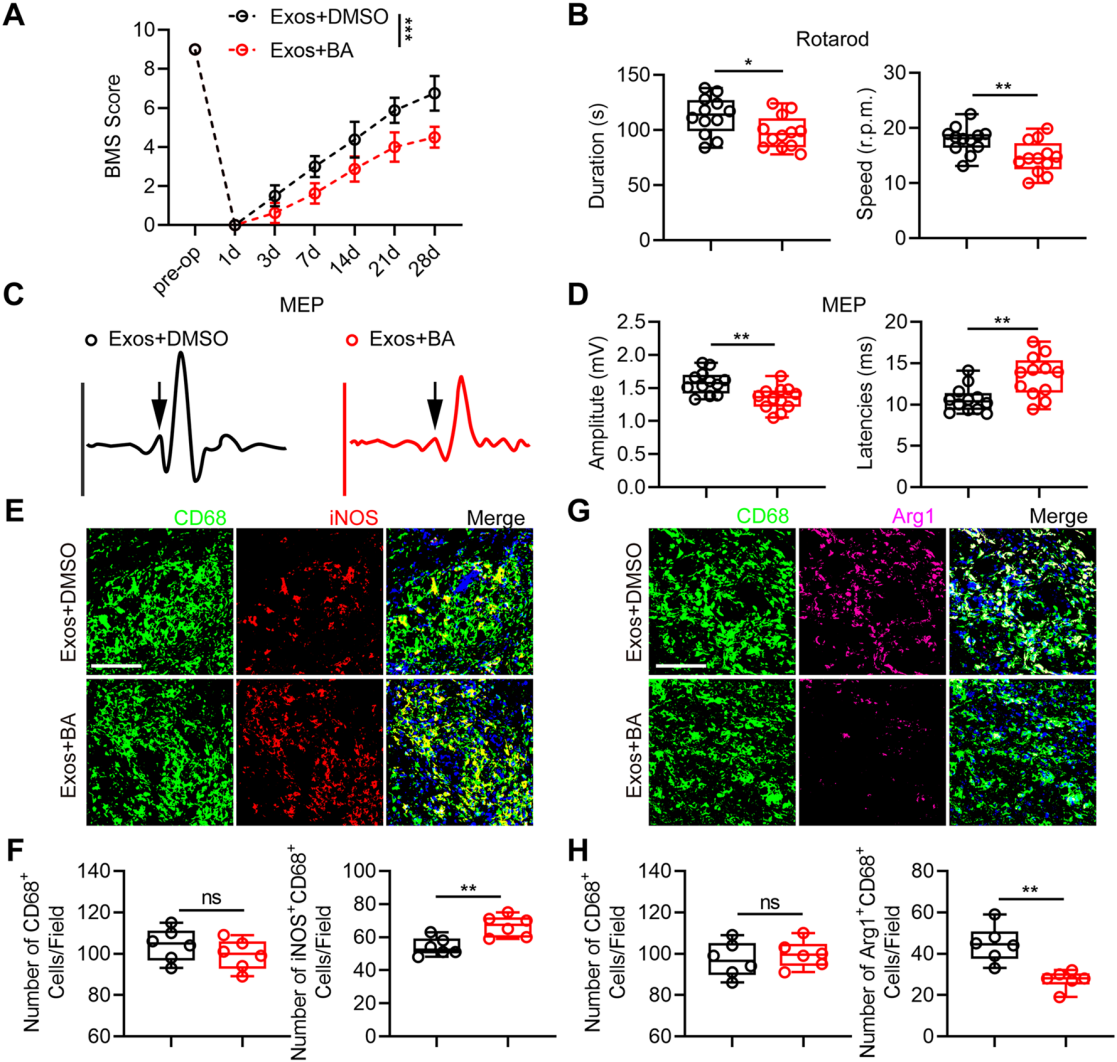
Exos treatment promotes functional rehabilitation and M2 polarization of microglia/macrophages through stabilizing IκBα thus inhibiting NF-κB signaling activation in vivo. **a** BMS scores of Exos-administrated mice which were treated with DMSO or BA, a selective inhibitor of IκBα. ***P < 0.001. **b** Evaluation of motor recovery by rotarod tests at day 28 after SCI. *P = 0.0302, **P = 0.0083. **c**, **d** Representative images and quantification of MEP amplitude and latencies in indicated groups at day 28 post-injury. **P < 0.01. **e**, **f** Double-staining of CD68 and iNOS at day 7 after SCI and quantification of CD68^+^ cells and M1 microglia/macrophages (Scale bar = 100 μm). **P = 0.0046. **g**, **h** Double-staining of CD68 and Arg1 at day 7 after SCI and quantification of CD68^+^ cells and M2 microglia/macrophages (Scale bar = 100 μm). **P = 0.0015

To further confirm the functional role of IκBα in Exos-mediated benefits in vivo, the selective inhibitor of IκBα BA was used. BA or vehicle (0.1% DMSO) was administered through the intralumbar post-injury. Functional in vivo analyses showed that administration of BA significantly suppressed motor function recovery of mice treated with Exos in comparison with administration of DMSO (Fig. 8a–d). Furthermore, qRT-PCR, western blot and immunofluorescence results revealed that administration of BA significantly suppressed microglia/macrophages M2 polarization in mice treated with Exos compared to those administered with DMSO (Additional file 1: Fig S7a–c and Fig. 8e–h). These findings indicate that Exos promote functional rehabilitation and M2 polarization of microglia/macrophages via upregulating IκBα and subsequently inhibiting NF-κB signaling pathway in vivo.

## Discussion

SCI causes irreversible motor and sensory disabilities with high mortality. BSCB is disrupted immediately after SCI resulting in subsequent spinal edema, neuroinflammation, neuronal cell death and glial activation [5, 6, 26]. Moreover, macrophages infiltrate into the central lesion after SCI. Infiltrated macrophages and the activated microglia are highly sensitive to SCI microenvironment and can be polarized to either M1 or M2 phenotype [9]. Therefore, it is essential to explore the underlying mechanism of the cell-to-cell communication in the SCI microenvironment and augment microglia/macrophages M2 polarization to inhibit detrimental neuroinflammation. In the present study, our results demonstrated that exosomes derived from vascular endothelial cells improve functional recovery and augment microglia/macrophages M2 polarization in vivo and in vitro. Notably, USP13 was highly upregulated in Exos and microglia/macrophages treated with Exos. Knockdown of USP13 in Exos reversed the beneficial functional effects of Exos. USP13 positively regulates microglia/macrophages polarization via suppressing ubiquitination-mediated degradation of IκBα. Addition of selective IκBα inhibitor BA abrogated the effects of Exos in vivo. These findings indicate a potential underlying mechanism in cell-to-cell communications between vascular endothelial cells and microglia/macrophages in SCI microenvironment. Moreover, the current study shows that exosomes derived from vascular endothelial cells are a promising therapeutic way for treating SCI and elucidates the underlying mechanisms.

Therapeutic effects of exosomes in promoting tissue regeneration as well as treating several disorders have been reported previously. Moreover, exosomes can cross the BBB easily and are promising agents in treating CNS diseases owing to their unique advantages including nano-sized and membrane-permeable characteristics [13, 27]. In addition, previous studies have explored the potential of using exosomes as genetic material or drug delivery vectors across BBB [28–30]. Previous studies on CNS injury reported that exosomes of stem cells remarkedly promote functional recovery after SCI, brain injury and stroke in mice [13, 17, 27, 31, 32]. Moreover, exosomes exhibited protective effect in treatment of myocardial infarction, cardiomyopathy and ischemia-reperfusion injury in nonhuman primates and preclinical trails [33–36]. Notably, clinical trial was performed to explore role of exosomes in the potential therapy of type I diabetes mellitus. In fact, the first patient with graft versus host disease was effectively treated with exosomes [37].

Microglia/macrophages play a dual role after SCI by exhibiting pro-inflammatory and anti-inflammatory phenotypes. Excessive M1 microglia/macrophages activation induces production of high-level proinflammatory cytokines resulting in severer chronic inflammation [9]. Therefore, recent publications have reported several approaches including pharmaceutic administration, gene therapy, MSCs transplantation and exosomes injection to regulate microglia/macrophages polarization [38]. This present study showed that conditional medium from vascular endothelial cells promotes M2 polarization, reduces ROS production and regulates mitochondrial function in BV2 microglia as well as RAW264.7 macrophages. Notably, these beneficial effects were significantly inhibited by the presence of GW4869 which is an exosomal secretion inhibitor. In vitro analyses showed that exosomes from endothelial cells increase microglia/macrophages M2 polarization and exosome-based signals are essential in crosstalk between vascular endothelial cells and microglia/macrophages. Furthermore, in vivo analysis showed that administration of exosomes improves functional recovery after SCI. Moreover, the potential mechanism in exosomes-based therapy in SCI were explored. Exosomes contain mRNAs, proteins and miRNAs which can be transferred to target cells and play their corresponding biological effects [39]. A recent study reported that LCP1 derived from BMSCs can be packed into exosomes and transmitted to osteosarcoma cells, thus promoting tumorigenesis and metastasis [40]. In addition, serum exosomes contain ECRG4 could suppress tumor growth [41]. Lv et al. reported that exosomal CCL2 from tubular epithelial cells plays an important role in tubulointerstitial inflammation [21]. Moreover, exosomal CCL2 mRNA is a biomarker of active histological injury in IgA nephropathy [42]. Therefore, RNA-seq was performed in BV2 microglia treated with Exos or PBS. The findings showed that USP13 is highly upregulated in microglia treated with Exos. Furthermore, downregulation of USP13 in Exos partially abrogated the beneficial effects of Exos, which indicates that Exos-mediated favorable effects in treatment of SCI through transferring USP13.

Due to the presence of deubiquitinating enzymes (known as DUBs), ubiquitination is a reversible process. DUBs can cleave ubiquitin molecules from modified proteins. It has been reported that microglia/macrophages polarization is regulated by ubiquitination [15]. A recent study showed that USP19/NLRP3 axis shift macrophages polarization to M2 and thus promote anti-inflammatory response [43]. Wang et al. reported M2 macrophages polarization was inhibited by suppressing Ubc9 [44]. Another study proved that USP10 promotes M2 macrophages polarization by deubiquitinating NLRP7 [45]. Furthermore, other studies reported that USP8, A20 as well as PHLDA1 regulate microglia polarization through different mechanisms [46–48]. This present study showed that USP13 was markedly increased in Exos and regulated the polarization of macrophages/microglia. USP13 is a member of the DUBs family and plays key roles in various biological processes including tumor promotion, inflammation, apoptosis, drug resistance and anti-viral responses by cleaving ubiquitin molecules from associated proteins including STING, Myc, PTEN and Mcl-1 [49–52]. However, the detailed function of USP13 in neuroscience, mainly in regulating macrophages/microglia polarization after CNS injury has not been fully explored.

To investigate the potential mechanisms underlying the effects of USP13 on regulating M2 microglia/macrophages polarization, IP/MS was performed and IκBα was identified as a potential target that binds to USP13 in microglia. IκBα is a subunit in the IKK complex. Transcriptional activation of NF-κB occurs when the IKK complex is activated and IκB proteins are phosphorylated. IκBα strongly binds to and sequesters NF-κB in the cytoplasm of resting cells. Stimulation of the IκBα kinase/IKK complex promotes phosphorylation of serine 32/36 of IκBα. Phosphorylation of IκBα promotes degradation of IκBα and releases NF-κB, which translocates to the nucleus [53–55]. Previous studies reported several proteins which target and control degradation and stability of IκBα. For example, Ji et al. reported that TRIM22 is implicated in promoting degradation of IκBα, thus activating NF-κB signaling [56]. A previous study reported that β-TrCP1 is involved in proteasomal-mediated degradation of IκBα [57]. Li et al. reported that USP34 inhibits NF-κB signaling activation by stabilizing IκBα [58]. In the present study, a novel DUB named USP13 which stabilizes IκBα was identified. The findings showed that USP13 interacts with IκBα and inhibits the ubiquitination of IκBα, leading to reduced degradation of IκBα in vitro and in vivo. Moreover, administration of IκBα inhibitor in vivo abolished M2 microglia/macrophages polarization resulted from Exos administration, suggesting that the favorable benefits of Exos on regulating microglia/macrophages polarization depends on the stability of IκBα. In all, this study suggests that USP13 regulates the polarization of microglia/macrophages, in part through promoting IκBα stability.

## Conclusion

In summary, this current study uncovered that exosomes derived from vascular endothelial cells improve functional recovery and augment microglia/macrophages M2 polarization via delivering USP13, which subsequently inhibits IκBα ubiquitination and degradation (Additional file 1: Fig. S8). This study provides a potential underlying mechanism in cell-to-cell communication between vascular endothelial cells and microglia/macrophages and show that exosomes are a promising approach for SCI treatment.

## Supplementary Information

**Additional file 1.** Additional figures.

## Data Availability

All the data supporting the conclusions of this article are included within this article and the additional files. The data used or analyzed during the current study are available on reasonable request.
