# Supplementary material for: Exosomal USP13 derived from microvascular endothelial cells regulates immune microenvironment and improves functional recovery after spinal cord injury by stabilizing IκBα
Source: Cell Biosci. 2023 Mar 13;13:55. doi: 10.1186/s13578-023-01011-9 (PMC10012460; doi:10.1186/s13578-023-01011-9)
Supplement: Supplementary file 1 — Additional file 1. Additional figures. [file 13578_2023_1011_MOESM1_ESM.docx]

**Supplementary Figures and Figure legends**

**
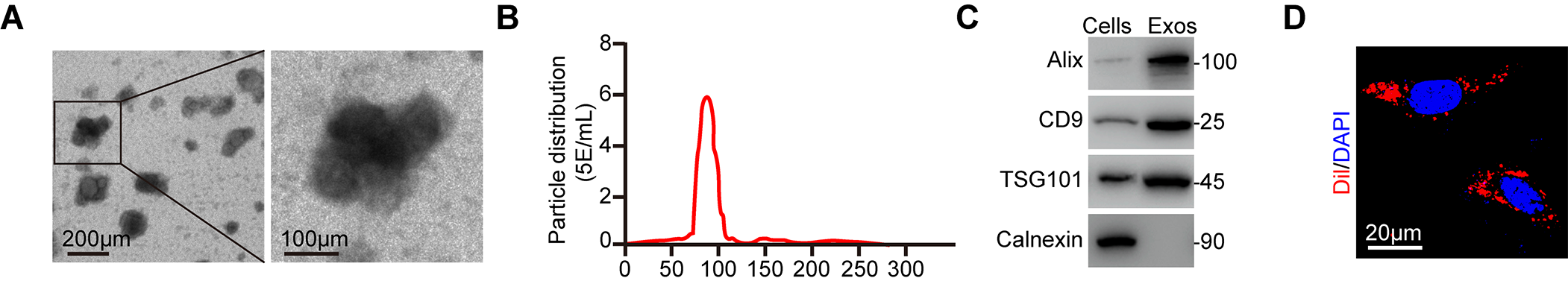
**

**Fig. S1** Identification and characterization of exosomes derived from vascular endothelial cells. (a) Morphology of exosomes derived from vascular endothelial bEnd.3 cells as observed under TEM. (b) NTA analysis showing particle distribution of exosomes. (c) Western blot analysis of biomarkers of exosomes including Alix, CD9 and TSG101 and the negative control Calnexin. (d) Uptake of red fluorescence dye Dil-labeled exosomes into BV2 microglia.


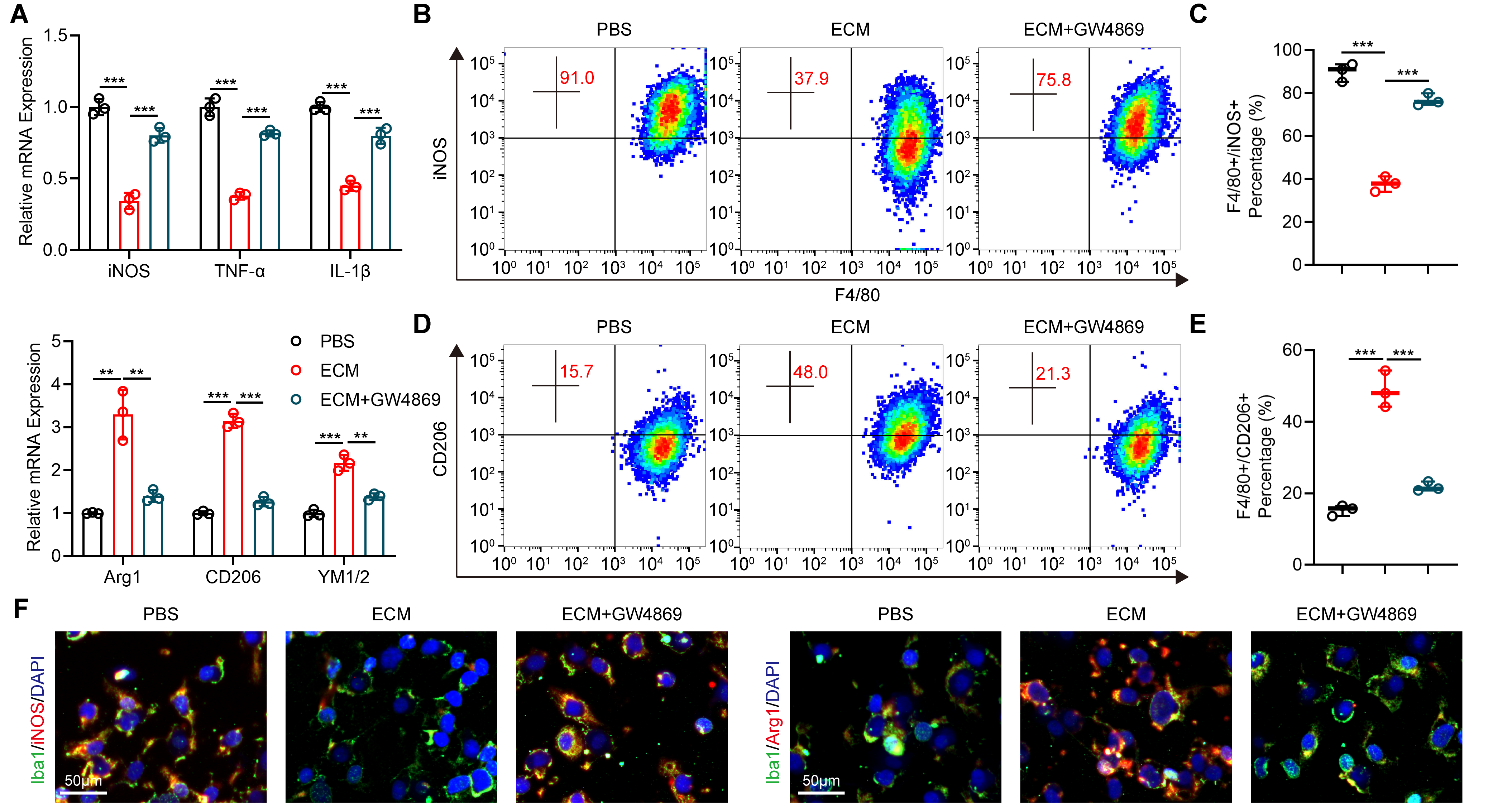


**Fig. S2** Exos shift RAW264.7 macrophages and primary microglial cells to M2-like polarization in response to LPS *in vitro*. (a) mRNA expression levels of M1-like and M2-like markers in RAW264.7 macrophages in indicated groups. **P ＜ 0.01, ***P ＜ 0.001. (b) Flow cytometry analysis of RAW264.7 macrophages in indicated groups. Dots represent F4/80 and iNOS staining. (c) Percentages of M1-like (F4/80^+^ and iNOS^+^) RAW264.7 macrophages. ***P ＜ 0.001. (d) Flow cytometry analysis of RAW264.7 macrophages in indicated groups. Dots represent F4/80 and CD206 staining. (e) Percentages of M2-like (F4/80^+^ and CD206^+^) RAW264.7 macrophages. ***P ＜ 0.001. (f) Representative immunostaining images of iba1, iNOS, and Arg1 in indicated groups of primary microglial cells.


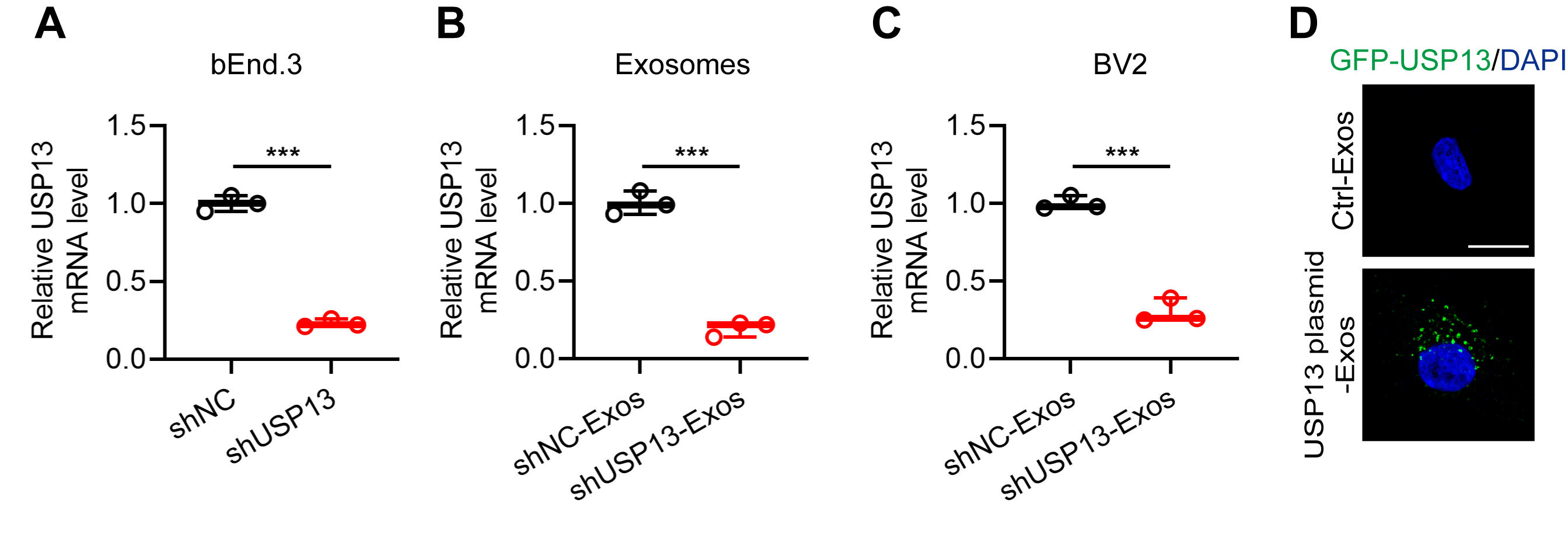


**Fig. S3** Exosomal transfer of USP13 from bEnd.3 cells to BV2 microglia. (a) Transfection efficiency of shUSP13 in bEnd.3 cells. ***P ＜ 0.001. (b) mRNA expression levels of USP13 in shNC-Exos and shUSP13-Exos. ***P = 0.0001. (c) mRNA expression levels of USP13 in BV2 microglia treated with shNC-Exos and shUSP13-Exos. ***P = 0.0002. (d) The bEnd3 cells were transfected with GFP-tagged USP13 plasmid or empty vector. Fluorescence images of BV2 microglia after treatment with exosomes derived from bEnd.3 cells (Scale bar = 50 μm).


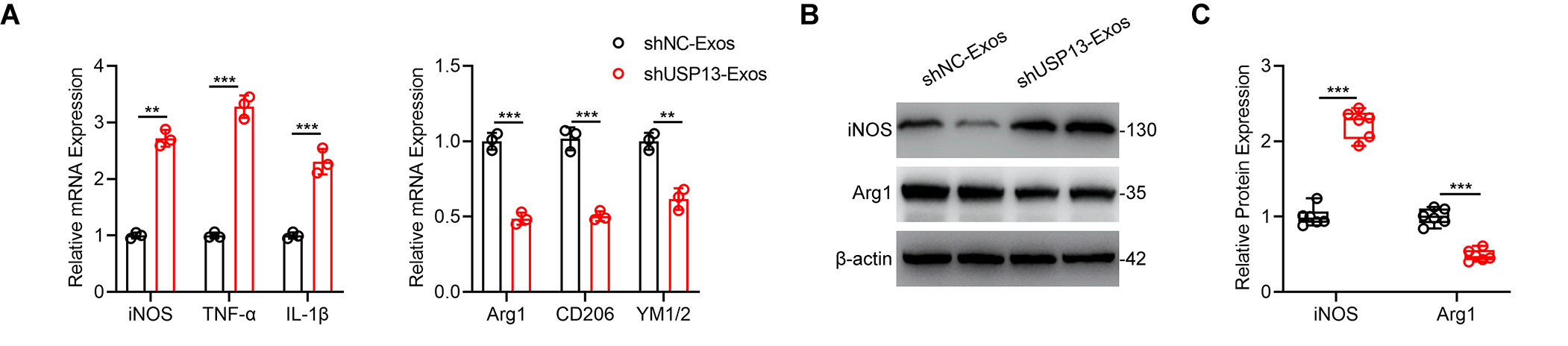


**Fig. S4** Administration of Exos shift microglia/macrophages to M2-like polarization after SCI by upregulating USP13 *in vivo*. (a) mRNA expression levels of M1-like and M2-like markers in the injured spinal cord from indicated groups at day 7 post-injury. **P ＜ 0.01, ***P ＜ 0.001. (b, c) Protein expression level of iNOS and Arg1 as determined by western blot analysis at day 7 post-injury. ***P ＜ 0.001.


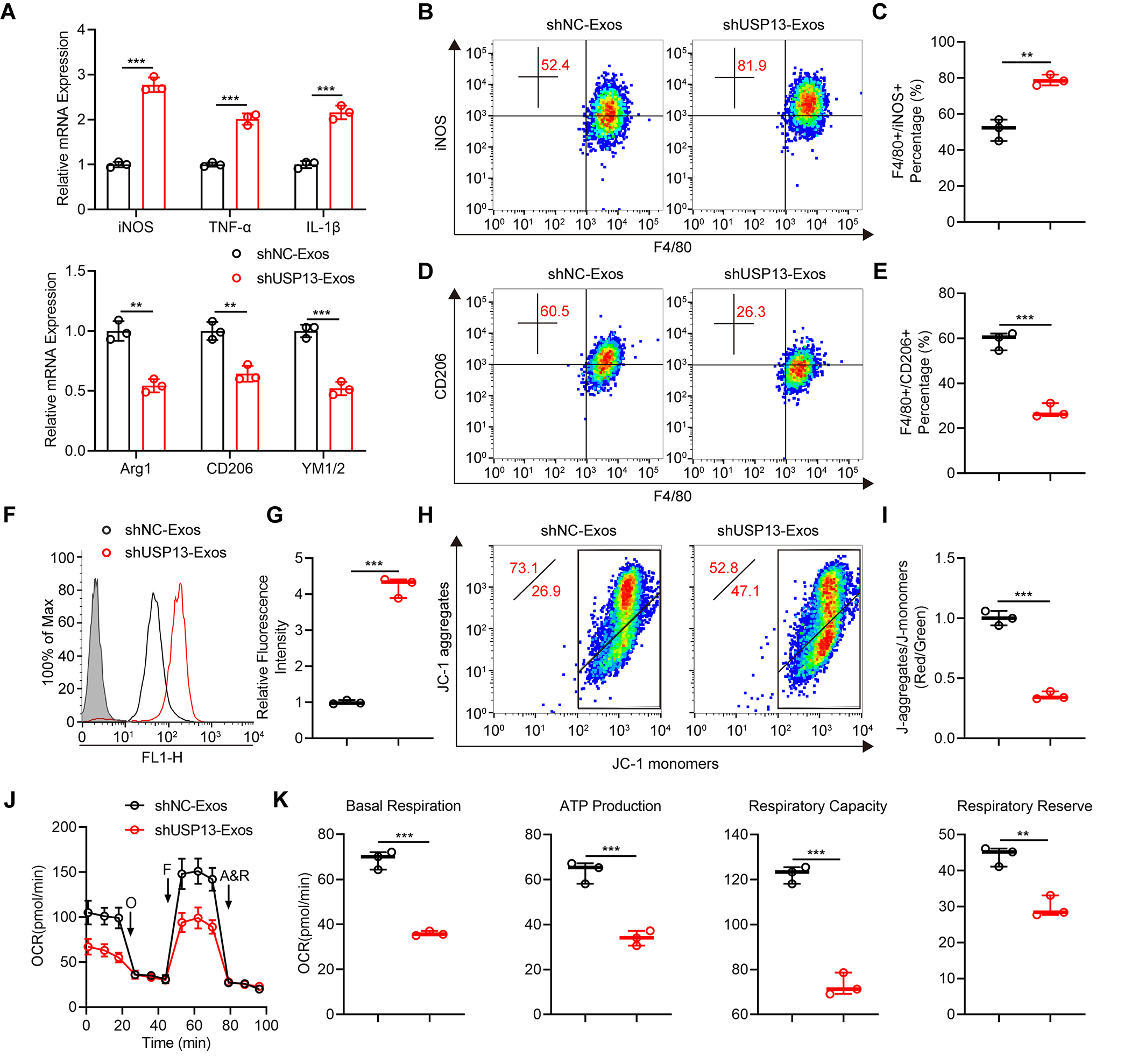


**Fig. S5** Exos treatment regulates microglia M2 polarization and modulates mitochondrial function through transferring USP13 *in vitro*. (a) Detection of mRNA levels of M1 and M2 markers in indicated groups. **P ＜ 0.01, ***P ＜ 0.001. (b, c) Measurement and quantification of M1 microglia polarization by flow cytometry. **P = 0.21. (d, e) Analysis and quantification of M2 microglia polarization by flow cytometry. ***P = 0.0004. (f, g) Flow cytometry detection and analysis of ROS. ***P ＜ 0.001. (h, i) Detection and quantification of mitochondrial potential by JC-1 staining. ***P ＜ 0.001. (j) Measurement of OCR. (k) Quantification of mitochondrial activities. **P = 0.0033, ***P ＜ 0.001.


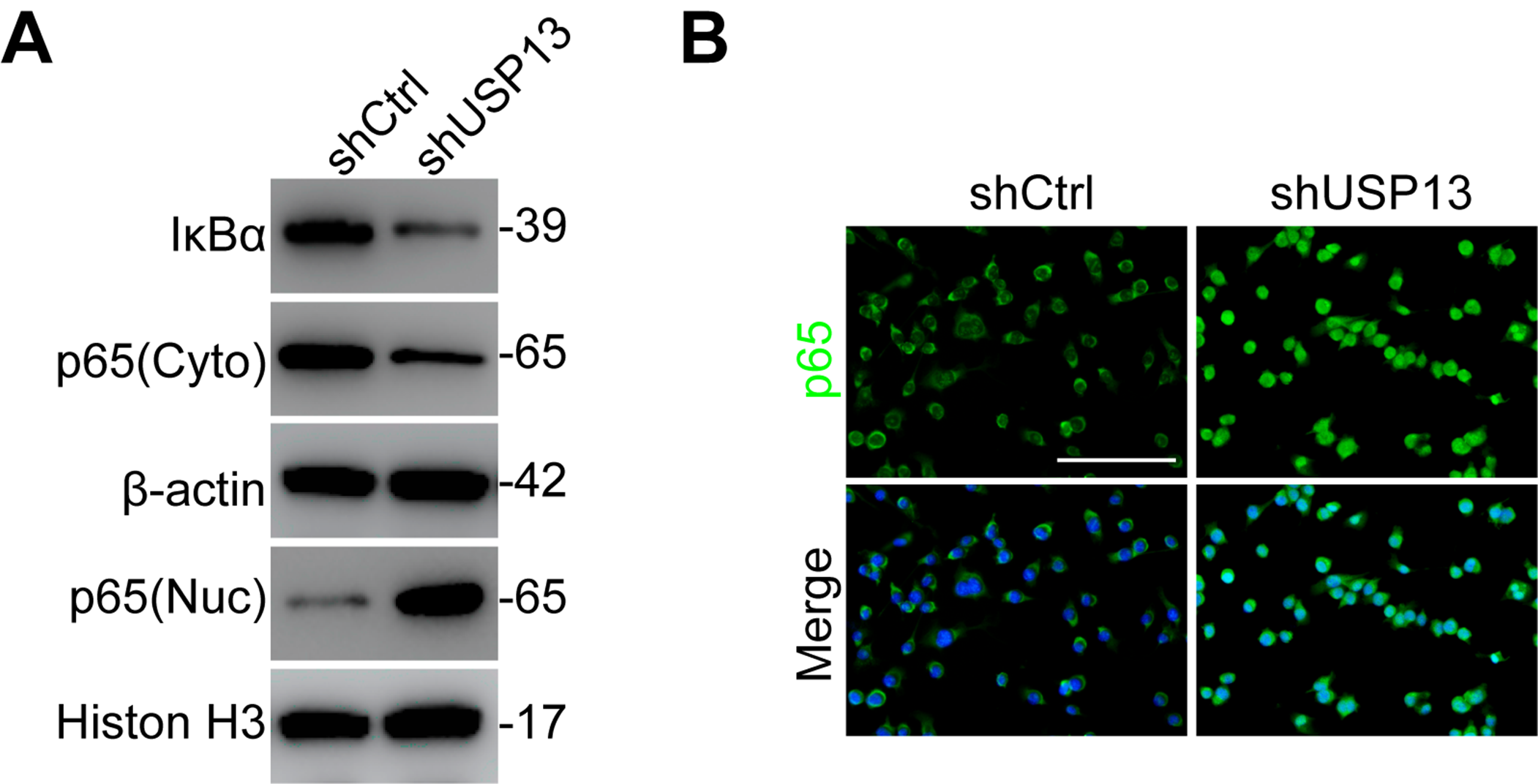


**Fig. S6** Knockdown of USP13 decreases IκBα protein level and activates NF-κB signaling in BV2 microglia. (a) Protein expression levels of IκBα, p65 (cytoplasm) and p65 (nucleus) in indicated groups in BV2 microglia as determined by western blot. (b) Immunofluorescence for p65 showing cellular localization in indicated groups in BV2 microglia.


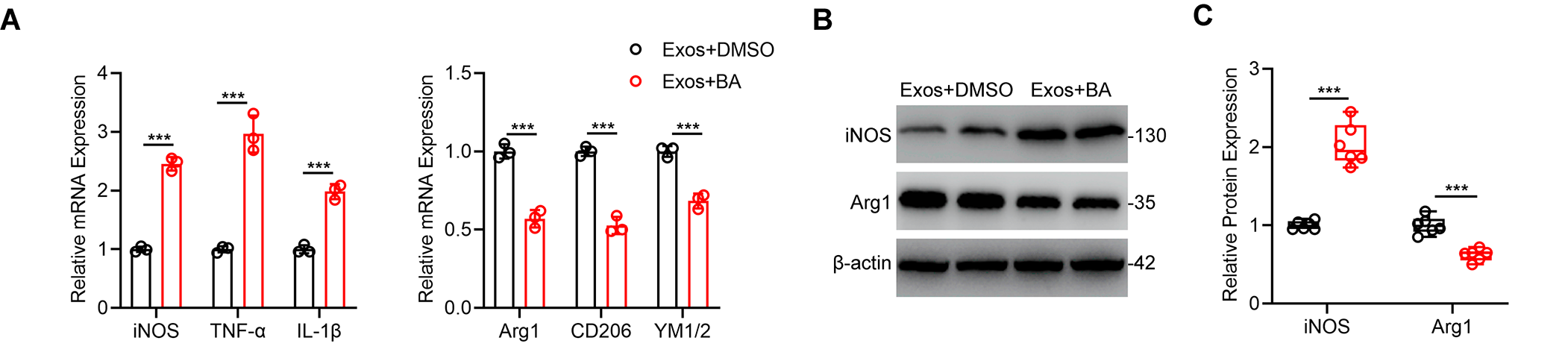


**Fig. S7** Exos promote functional recovery and shift microglia/macrophages to M2-like phenotype by stabilizing IκBα thus inhibiting activation of NF-κB signaling *in vivo*. (a) mRNA expression levels of M1-like and M2-like markers in injured spinal cord from different groups at day 7 post-injury. ***P ＜ 0.001. (b, c) Protein expression level of iNOS and Arg1 as determined by western blot at day 7 post-injury. ***P ＜ 0.001.


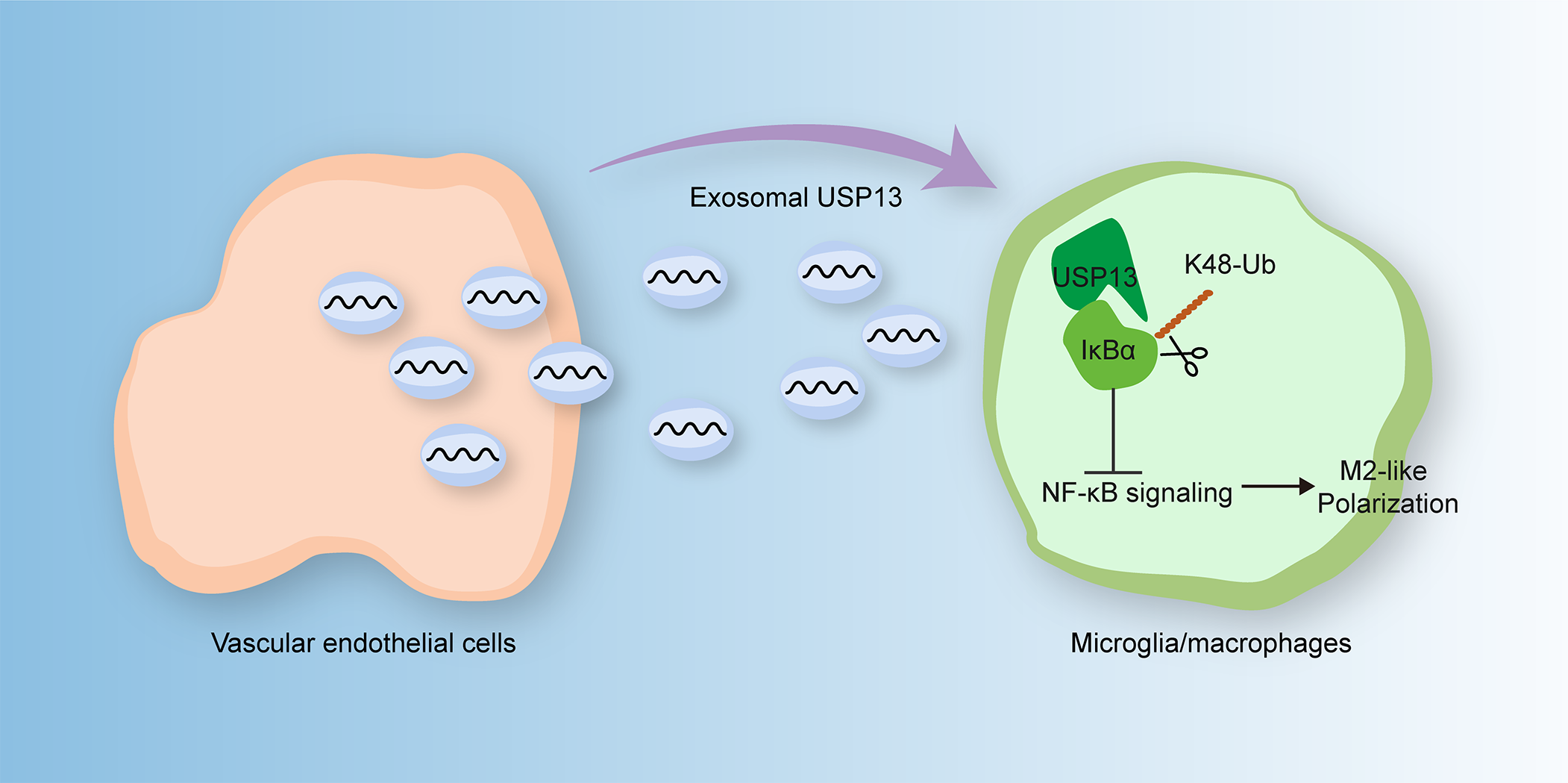


**Fig. S8** Potential underlying mechanism by which exosomal USP13 derived from vascular endothelial cells promotes microglia/macrophages M2 polarization. Exosomes derived from vascular endothelial cells shift microglia/macrophages towards M2 polarization and regulate mitochondrial function via transferring USP13, which subsequently inhibits IκBα ubiquitination and degradation, and ultimately suppresses NF-κB signaling, resulting in better functional recovery after spinal cord injury.
